# Increased Horizontal Transmission of Recombinant Marek’s Disease Virus Due to Reticuloendotheliosis Virus Long Terminal Repeat Is the Major Competitive Advantage of the Virus Being a Prevalent Strain

**DOI:** 10.3389/fmicb.2019.02842

**Published:** 2019-12-13

**Authors:** Shuai Su, Ning Cui, Yanpeng Li, Meng xin Yu, Ting Zhang, Ziqiang Cheng, Jiaqian Chai, Zhizhong Cui, Ruiai Chen

**Affiliations:** ^1^College of Veterinary Medicine, Shandong Agricultural University, Tai’an, China; ^2^Shandong Provincial Key Laboratory of Animal Biotechnology and Disease Control and Prevention, Shandong Agricultural University, Tai’an, China; ^3^Shandong Key Laboratory of Animal Disease Control and Breeding, Institute of Animal Science and Veterinary Medicine, Shandong Academy of Agricultural Sciences, Jinan, China; ^4^Zhaoqing Institute of Biotechnology Co., Ltd., Zhaoqing, China; ^5^College of Veterinary Medicine, South China Agricultural University, Guangzhou, China

**Keywords:** Marek’s disease virus, REV *LTR*, horizontal transmission, competitive advantage, virus gene transcription

## Abstract

GX0101 is the first field Marek’s disease virus (MDV) recombinant with an REV *LTR* insert isolated in China. We speculated that there was a selective advantage of GX0101 becoming the more prevalent field strain from a very low percentage of recombinant virus. In the study, dual fluorescence quantitative real-time PCR (DF-qPCR) that detects GX0101 and GX0101Δ*LTR* simultaneously was established based on the genomic difference of GX0101 and its *LTR* deletion strain GX0101Δ*LTR*. MDV natural transmission was simulated in specific-pathogen-free (SPF) chicks, and continuous tracking of GX0101 and GX0101Δ*LTR* in chicks was carried out. The results showed that GX0101 possessed high horizontal transmission capacity, which could infect SPF chicks by contact in a short time and became the predominant strain following contact infections in chicken flocks. GX0101 still had a more significant advantage of horizontal transmission than GX0101Δ*LTR* after continuous passage even if the initially infectious dose was significantly lower. There were 72 differentially expressed MDV genes between GX0101 and GX0101Δ*LTR*, with the genes and gene products mainly involved in virus replication, tegument protein, glycoprotein, nucleocapsid protein, immune evasion, tumor development and/or pathogenesis, and hypothetical protein. Sixteen genes related to virus replication and transmission were significantly up-regulated. This is the first study to illuminate that increased horizontal transmission of recombinant MDV due to REV *LTR* was the competitive advantage of the virus being a prevalent strain and define the differential transcription profile of viral genes between GX0101 and GX0101Δ*LTR*. This will be helpful for in-depth study on the molecular mechanism of increased horizontal transmission of MDV by REV *LTR*.

## Introduction

Marek’s disease (MD), induced by the Marek’s disease virus (MDV), is a contagious lymphoproliferative disease of poultry (Churchill and Biggs, 1967). GX0101 is the first natural recombinant MDV field strain isolated from birds showing tumors in China (Cui et al., 2010). We constructed an infectious bacterial artificial chromosome (BAC) clone of GX0101, which showed characteristics similar to those of the parental virus in replication and pathogenicity (Sun et al., 2009). The complete genome of GX0101 was sequenced and analyzed using the GX0101 BAC clone (Su et al., 2012, 2013). It contains a 538-bp reticuloendotheliosis virus (REV) long terminal repeat (*LTR*) inserted between nucleotide bases “C” and “A” numbered 153,175–153,176 (Md5 strain) or 154,507–154,508 (RB1B strain). GX0101 is a very virulent MDV, with greater horizontal transmission ability than Md5 (Xu et al., 2009), while other reported recombinant MDV strains with an REV *LTR*, such as RM1 obtained from cell cultures, are attenuated and do not cause tumors (Witter et al., 1997; Sun et al., 2010). MDV GX0101Δ*LTR*, an *LTR*-null strain of GX0101, was produced by the RedE/T recombination technology. An flp recognition target (FRT) site of 84 bp in length remained in the genome of GX0101Δ*LTR* (Sun et al., 2010).

As the rate of such integration events is usually considered to be low, the isolation of the GX0101 strain of chimeric virus would suggest that the integration of REV *LTR* might provide some selective advantages in replication for such viruses that resulted in their ready isolation from the infected birds. In this study, we simulated the natural transmission of MDV via continuous contact infections of MDV in specific-pathogen-free (SPF) chickens to study the major competitive advantage of MDV being a prevalent strain.

## Materials and Methods

### Cell Cultures and Viruses

Specific-pathogen-free chicks and chicken embryos for the preparation of chicken embryo fibroblast (CEF) cultures were obtained from SPAFAS Co. (Jinan, China). The CEF cultures were used for virus propagation, virus reactivation assays, and DNA transfections. Infectious BAC-derived GX0101Δ*LTR* virus was previously rescued by transfection of BAC DNA into CEF cultures (Sun et al., 2010).

### Primers and Probes for Detection of GX0101 or GX0101Δ*LTR* by Dual Fluorescence Quantitative Real-Time PCR (DF-qPCR)

**FIGURE 1:**
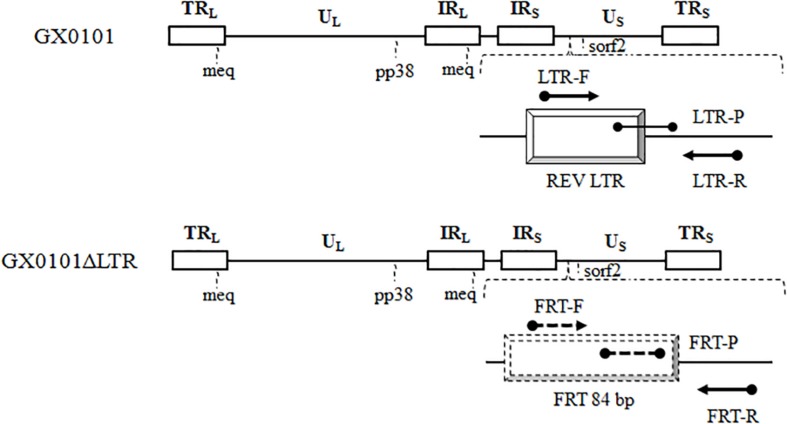
Primers and probes for detection of GX0101 or GX0101Δ*LTR* by DF-qPCR. LTR is located upstream of the sorf2 gene in the GX0101 genome. There is an 84-bp length of FRT sites residue in the GX0101Δ*LTR* genome after deleting LTR, which is the unique difference between GX0101Δ*LTR* and GX0101. *LTR*-F/R: *LTR*-F/R primers; *LTR*-P: *LTR*-P probe; FRT-F/R: FRT-F/R primers; FRT-P: *LTR*-P probe.

The probe detecting GX0101 is located in the chimeric area of REV *LTR*, and the probe detecting GX0101Δ*LTR* is in the FRT residue. Forward primers of two independent qPCR of DF-qPCR are different, and reverse primers are the same. This is effective in identifying the two different MDVs without mutual interference (Figure 1 and Table 1).

**TABLE 1:** Oligonucleotide primers and probes used in DF-qPCR.

**Primers/probes**	**Sequences (5′–3′)**	**Primer location**	**PCR product**	**Target**
LTR-F	GGTAGGGATCCGGACTGAATC	153218–153238	123 bp	GX0101
LTR-R	GAGGATGCATATGTCGCAACA	153320–153340		
LTR-P	CGGTACAACAACCATCAA (5′-FAM, 3′-TAMRA)	153249–153266		
FRT-F	TGATGGTCATTCCGGGGAT	–	164 bp	GX0101Δ*LTR*
FRT-R	GAGGATGCATATGTCGCAACA	–		
FRT-P	CGACGGATCCCCGGAA (5′-Hex, 3′-TAMRA)	–		

### Reaction of DF-qPCR

DF-qPCR reactions were set up on ice, and each reaction contained the following: *LTR*-F/R primers, FRT-F/R primers (all at 0.25 μM), 5-carboxyfluorescein (FAM)-fluorescent-tagged *LTR* probe and (Hex)-fluorescent-tagged FRT probe (both at 0.1 μM), 10 μl 2 × TaqMan^®^ Gene Expression Master Mix buffer, and 2 μl of DNA. The reaction volume was brought up to 20 μl by the addition of water. An ABI PRISM^®^ 7500 sequence detection system (Applied Biosystems) was used to amplify and detect the reaction products under the following conditions: 50°C for 2 min and 95°C for 10 min, followed by 40 cycles of 94°C (15 s) and 60°C (1 min).

### Specificity of DF-qPCR in Detecting GX0101 or GX0101Δ*LTR*

The specificity of GX0101 or GX0101Δ*LTR* single fluorescence quantitative real-time PCR (SF-qPCR) was firstly detected. To analyze the specificity of GX0101Δ*LTR* SF-qPCR, the 20-μl PCR reactions contained 2 μl (200 pg) of GX0101Δ*LTR* DNA, 0.1 μM FRT-P probe, and 0.25 μM FRT-F/R primers, and 10 μl 2 × TaqMan^®^ Gene Expression Master Mix buffer was used as standard reaction system. Additional 2 μl (200 pg) of GX0101 DNA or 0.1 μM *LTR*-P probe and 0.25 μM *LTR*-F/R primers were added in the reaction system. ABI PRISM^®^ 7500 sequence detection system (Applied Biosystems) was employed to detect the Ct value of GX0101Δ*LTR*. Interference of GX0101Δ*LTR* DNA or FRT-F/R and FRT-P in the GX0101 SF-qPCR detection system was also determined according to the above method.

After verification of the specificity of SF-qPCR in detecting GX0101 or GX0101Δ*LTR*, the specificity of DF-qPCR in detecting GX0101 or GX0101Δ*LTR* was analyzed. The 20-μl PCR reactions contained 2 μl of GX0101Δ*LTR* DNA and GX0101Δ*LTR* DNA, 0.1 μM FRT-P probe and *LTR*-P probe, 0.25 μM FRT-F/R primers, and *LTR*-F/R primers, and 10 μl 2 × TaqMan^®^ Gene Expression Master Mix buffer was used as standard reaction system. ABI PRISM^®^ 7500 sequence detection system (Applied Biosystems) was employed to detect the Ct of GX0101Δ*LTR* and GX0101 in the DF-qPCR reaction system.

### Establishment of Standard Curve

BAC plasmid of GX0101 and GX0101Δ*LTR* was prepared using the QIAGEN kit and quantified with ultraviolet spectrophotometry. The plasmid was diluted to 10^9^ copies per 2 μl and serially diluted to 10 copies per 2 μl by 10 times gradient dilution. The Ct value of GX0101 BAC and GX0101Δ*LTR* BAC from 10^9^ to 10^1^ copies was detected and the standard curve was established on the ABI PRISM^®^ 7500 sequence detection system.

### Horizontal Transmission Capacity of GX0101 and GX0101Δ*LTR* in SPF Chicks Inoculated With the Same Dose

Horizontal transmission capacity of GX0101 and GX0101Δ*LTR* was compared via successive contact infections by simulating MDV natural transmission in SPF chicks (Supplementary Figure 1). Thirty 1-day-old SPF chicks were raised in one isolator. Ten chicks were inoculated with 1000 plaque-forming-units (PFU) of GX0101 by the intra-abdominal (IA) route, and another 10 chicks were inoculated with 1000 PFU of GX0101Δ*LTR*. The surplus 10 chicks were used as contact ones. All chicks were marked respectively. Viral copies of GX0101 and GX0101Δ*LTR* in the contact chicks were determined by detecting the DNA of the feather tips using DF-qPCR at 7, 14, 21, and 28 days post-inoculation, respectively. Briefly, six to eight pieces of 1- to 2-mm-long feather tips were collected from each bird. They were incubated in 0.5 ml of digestion buffer (100 mM NaCl, 10 mM Tris–HCl, pH 8.0, 0.25 mM EDTA, 0.5% SDS, and 100 μg/ml proteinase K) overnight at 55°C. DNA in solution was extracted by phenol/chloroform mixture and then precipitated with alcohol, and dissolved into 50 μl of TE buffer (10 mM Tris–HCl and 0.1 mM EDTA, pH 8.0).

At 28 days post-inoculation, the 10 chicks for contact infection of MDV were moved to another isolator. Another ten 1-day-old SPF chicks were put in the isolator for the next round of contact infection at the same time. Viral copies in the second round of contact chicks were also determined after 21 and 28 days cohabitation, respectively. After 28 days cohabitation, the 10 chicks for the second round of contact infection were moved to another isolator. Another ten 1-day-old SPF chicks were put in the isolator for the third round of contact infection. Viral copies in the third round of contact chicks were then determined after 21 and 28 days cohabitation, respectively.

### Horizontal Transmission Capacity of GX0101 and GX0101Δ*LTR* in SPF Chicks Inoculated With Different Doses

Fifteen SPF chicks were inoculated with 100 PFU of GX0101 or 2000 PFU of GX0101Δ*LTR* by the IA route (Supplementary Figure 2). Ten cohabitant chicks were used for contact infection. Viral copies of the two viruses in the contact chicks were determined after 28 and 35 days cohabitation, respectively. After 35 days cohabitation, the 10 chicks for contact infection were moved to another isolator. Another ten 1-day-old SPF chicks were put in the isolator for the second round of contact infection at the same time. Viral copies in the second round of contact chicks were also determined after 28 and 35 days cohabitation, respectively. Sample collection and DNA extraction were carried out by the same method described above.

### Differential Expression of MDV Genes Between GX0101 and GX0101Δ*LTR*

Primary CEF cells collected from one embryo were seeded onto three individual flasks with the density of 5 × 10^6^ cells/flask. Cells in one of the flasks were infected with GX0101 (1.5 × 10^5^ PFU/flask), another flask was infected with GX0101Δ*LTR* (1.5 × 10^5^ PFU/flask), and the last one was mock-infected with Dulbecco’s Modified Eagle’s Medium (DMEM). Total RNA was extracted from GX0101- or GX0101Δ*LTR*-infected cells at 56 h post-infection using TRIzol Reagent (Life Technologies, Carlsbad, CA, United States) following the manufacturer’s instructions, and RNA integrity was checked using an Agilent Bioanalyzer 2100 (Agilent Technologies, Santa Clara, CA, United States). A 4 × 44 K Agilent custom oligo microarray (array ID: 042688) was employed to analyze the transcription profile of MDV viral genes in cell cultures. Four biological replicates were used in each group with dye balance. Transcription level of viral genes was compared between the two infectious groups. Data normalization was performed using locally weighted scatter plot smoothing (LOWESS) by R project^1^. The *P*-value and fold changes for each gene were calculated. A gene was considered to be significantly differentially expressed only if the log2 median of the ratios of the Cy5:Cy3 signal was greater than 1.00-fold or lower than −1.00-fold with *P* < 0.05. Five differentially expressed genes in the microarray analysis were verified by quantitative real-time RT-qPCR. RT-PCR analysis was performed using the ABI PRISM^®^ 7500 sequence detection system with SYBR Premix Ex Taq II (TaKaRa, China) following the manufacturer’s instructions. Glyceraldehyde-3-phosphate dehydrogenase (GAPDH) was used as the endogenous reference gene to normalize the reactions to the same amplification progression. After amplification, the relative fold change of the differentially expressed genes was calculated through the 2−^ΔΔCq^ method. Triplicate RT-qPCRs were performed on each cDNA to guarantee the reproducibility of the amplification.

## Results

### The SF-qPCR Has Specificity to Detect GX0101 or GX0101Δ*LTR*

**FIGURE 2:**
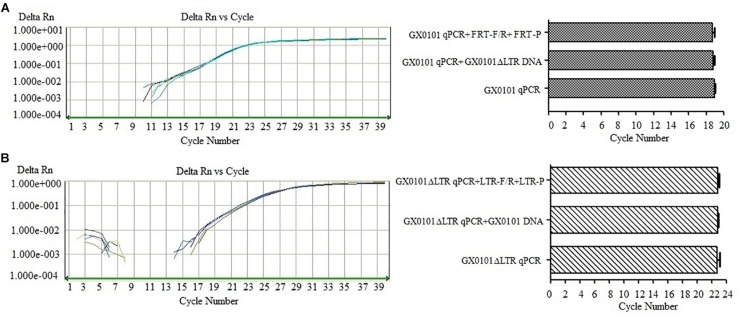
Results of SF-qPCR for detecting GX0101 **(A)** or GX0101Δ*LTR* **(B)**. **(A)** Results of SF-qPCR for detecting GX0101, with additional GX0101Δ*LTR* DNA or FRT-F/R primer and FRT-P probe in the reaction system. **(B)** Results of SF-qPCR for detecting GX0101Δ*LTR*, with additional GX0101 DNA or LTR-F/R primer and LTR-P probe in the reaction system.

The SF-qPCR for detecting GX0101 or GX0101Δ*LTR* with good specificity was established after the optimization of primers and probes. Detection of GX0101 by SF-qPCR was not affected by GX0101Δ*LTR* DNA or primers/probes of GX0101Δ*LTR* (Figure 2A). The amplification curves overlapped, and the Ct values were similar, with no significant differences (*P* > 0.05), when the GX0101Δ*LTR* DNA or primers/probes of GX0101Δ*LTR* were added in the SF-qPCR detecting system for GX0101. Similarly, the amplification curves overlapped, and the Ct values were similar, with no significant differences (*P* > 0.05), when the GX0101 DNA or primers/probes of GX0101 were added in the SF-qPCR detecting system for GX0101Δ*LTR* (Figure 2B). The results demonstrated good specificity of SF-qPCR for detection of GX0101 or GX0101Δ*LTR*, respectively.

### The DF-qPCR Has Specificity to Detect GX0101 and GX0101Δ*LTR* Simultaneously

**FIGURE 3:**
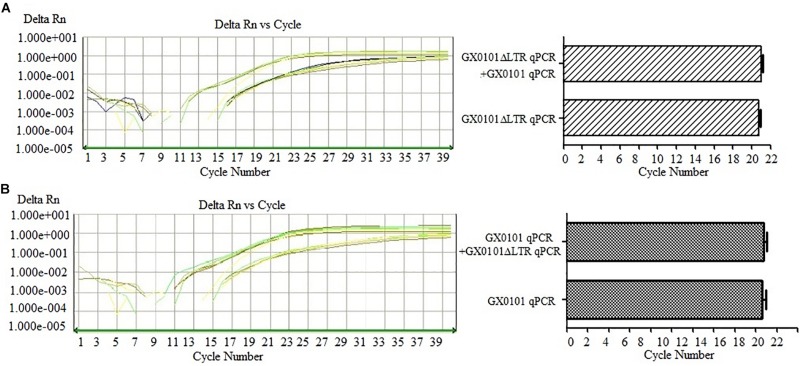
Results of DF-qPCR for detecting GX0101 **(A)** and GX0101Δ*LTR* **(B)**. **(A)** Comparison of SF-qPCR and DF-qPCR for detecting GX0101Δ*LTR*. **(B)** Comparison of SF-qPCR and DF-qPCR for detecting GX0101.

The DF-qPCR was used for detecting GX0101 and GX0101Δ*LTR* simultaneously in a single tube. Ct values of GX0101Δ*LTR* in the DF-qPCR system were similar to those in the SF-qPCR system that only detected GX0101Δ*LTR* (Figure 3A), with no significant differences (*P* > 0.05). Similarly, Ct values of GX0101 in the DF-qPCR system were also similar to those in the SF-qPCR system that only detected GX0101 (Figure 3B), with no significant differences (*P* > 0.05). The results demonstrated that the established DF-qPCR system showed good specificity, in which tube GX0101 and GX0101Δ*LTR* amplified respectively without interference with each other.

### Standard Curves of GX0101 and GX0101Δ*LTR*

The standard curve of samples can be automatically generated by the configured SDS.V1.4 software according to the procedure on the ABI PRISM^®^ 7500 sequence detection system. The established standard curves of GX0101 and GX0101Δ*LTR* were *y* = −3.45*x* + 42.32 and *y* = −3.68*x* + 44.74 (*y* = Ct, *x* = log copy), respectively (Supplementary Figure 3).

### GX0101 Has Significant Advantage of Horizontal Transmission Over GX0101Δ*LTR* When the Same Dose Was Inoculated in SPF Chicks

SPF chicks were inoculated with the same dose of GX0101 or GX0101Δ*LTR* at 1 day old, which represented the same transmission source (Supplementary Figure 4). One chick from the GX0101 or GX0101Δ*LTR*-infected group died at 4 weeks post-inoculation throughout the experimental periods. GX0101 was detectable in the first generation of contact chicks at 14 dpi, with the infection rate of 20%. GX0101Δ*LTR* could only be detected at 28 dpi in 30% of the chicks, while the infection rate of GX0101 was up to 60% at 28 dpi (Table 2). GX0101 was detectable in the second generation of contact chicks at both 21 and 28 dpi, with the infection rate of 90% and 100%, respectively. Only one chick was positive for GX0101Δ*LTR* at 28 dpi (Table 3). GX0101 was also detectable in the third generation of contact chicks at both 21 and 28 dpi, with the infection rate of 90 and 100%, respectively, while GX0101Δ*LTR* was not detectable in any chicks (Table 4). After three generations of successive contact transmissions, the infection rate of GX0101 increased from 60% of first generation to 100%, while that of GX0101Δ*LTR* decreased from 30 to 0%. Thus, the results showed that GX0101 infected chicks in a shorter time than GX0101Δ*LTR* and spread by contact in chicken flocks quickly, demonstrating the epidemic advantage of GX0101.

**TABLE 2:** Viral copies of GX0101 and GX0101Δ*LTR* in the first generation of chickens infected by horizontal transmission after different contact times.

**Contact**	**MDV strain**	**SPF chicken no.**									
		**1#**	**2#**	**3#**	**4#**	**5#**	**6#**	**7#**	**8#**	**9#**	**10#**
7 days	GX0101	–	–	–	–	–	–	–	–	–	–
	GX0101Δ*LTR*	–	–	–	–	–	–	–	–	–	–
14 days	GX0101	–	–	–	5888	–	1905	–	–	–	–
	GX0101Δ*LTR*	–	–	–	–	–	–	–	–	–	–
21 days	GX0101	–	537	–	53703	186	14125	173	257	–	–
	GX0101Δ*LTR*	–	–	–	–	–	–	–	–	–	–
28 days	GX0101	–	11481	–	165958	851	70794	4570	3019	–	–
	GX0101Δ*LTR*	10000	–	1995	–	–	–	–	4466	–	–

*“–” represents the detection results are negative; the digits represent the copy number of virus. 1#–10# indicate the serial number of contact chickens.*

**TABLE 3:** Viral copies of GX0101 and GX0101Δ*LTR* in the second generation of chickens infected by horizontal transmission after different contact times.

**Contact**	**MDV strain**	**SPF chicken no.**									
		**1#**	**2#**	**3#**	**4#**	**5#**	**6#**	**7#**	**8#**	**9#**	**10#**
21 days	GX0101	–	2691	851	295	209	162	977	1202	208	407
	GX0101Δ*LTR*	–	–	–	–	–	–	–	–	–	–
28 days	GX0101	141	5888	1659	1119	3019	794	4549	8851	398	1279
	GX0101Δ*LTR*	1380	–	–	–	–	–	–	–	–	–

*“–” represents the detection results are negative; the digits represent the copy number of virus. 1#–10# indicate the serial number of contact chickens.*

**TABLE 4:** Viral copies of GX0101 and GX0101Δ*LTR* in the third generation of chickens infected by horizontal transmission after different contact times.

**Contact**	**MDV strain**	**SPF chicken no.**									
		**1#**	**2#**	**3#**	**4#**	**5#**	**6#**	**7#**	**8#**	**9#**	**10#**
21 days	GX0101	4898	1380	12302	151	–	2344	3715	794	17378	363
	GX0101Δ*LTR*	–	–	–	–	–	–	–	–	–	–
28 days	GX0101	12302	19953	39810	912	224	7943	27542	1549	46773	2692
	GX0101Δ*LTR*	–	–	–	–	–	–	–	–	–	–

*“–” represents the detection results are negative; the digits represent the copy number of virus. 1#–10# indicate the serial number of contact chickens.*

### GX0101 Still Has a Significant Advantage of Horizontal Transmission Over GX0101Δ*LTR* When a Lower Dose Was Inoculated in SPF Chicks

The first generation of 10 contact chicks was detected for MDV in the DNA of feather follicle at 28 dpi (Supplementary Figure 5). Nine chicks were positive for GX0101, and six chicks were positive for GX0101Δ*LTR*, with the infection rate of 90 and 60%, respectively. Five chicks were co-infected with GX0101 and GX0101Δ*LTR*, and three chicks had higher viral copies of GX0101Δ*LTR* than GX0101. Four chicks were infected with GX0101 alone, and one chick was single-infected with GX0101Δ*LTR*. After contact with infection for 35 days, 10 chicks were infected with GX0101, and 8 chicks were infected with GX0101Δ*LTR*, with a positive rate of 100 and 80%. Viral copies of GX0101Δ*LTR* were higher than GX0101 in three chicks. Two chicks were positive for GX0101 alone (Table 5). When the second generation of contact chicks cohabited with the first generation of contact chicks for 28 days, nine chicks were infected with GX0101, and five chicks were infected with GX0101Δ*LTR*, with a positive rate of 90 and 50%. Five chicks were co-infected with both viruses, with two chicks having higher viral copies of GX0101Δ*LTR* than GX0101. Four chicks were infected with GX0101 alone. After contact infection for 35 days, 10 chicks were infected with GX0101, and 6 chicks were infected with GX0101Δ*LTR*, with a positive rate of 100 and 60%. Viral copies of GX0101Δ*LTR* were higher than GX0101 in three chicks. Four chicks were infected with GX0101 alone (Table 6). Thus, the results showed that GX0101 still possessed obvious epidemic advantage after two successive contact transmissions, with high viral copies in contact chicks, even when the initial inoculation dose was only 1/20 of GX0101Δ*LTR*.

**TABLE 5:** Viral copies of GX0101 and GX0101Δ*LTR* in the first generation of chickens infected by horizontal transmission after different contact times.

**Contact**	**MDV strain**	**SPF chicken no.**									
		**1#**	**2#**	**3#**	**4#**	**5#**	**6#**	**7#**	**8#**	**9#**	**10#**
28 days	GX0101	124	211	4863	276	657	440	–	275	502	411
	GX0101Δ*LTR*	–	687	939	237	–	–	153	–	645	2897
35 days	GX0101	27579	502	1462	702	337	980	502	258	3724	502
	GX0101Δ*LTR*	5416	828	828	324	443	–	5416	–	237	447

*“–” represents the detection results are negative; the digits represent the copy number of virus. 1#–10# indicate the serial number of contact chickens.*

**TABLE 6:** Viral copies of GX0101 and GX0101Δ*LTR* in the second generation of chickens infected by horizontal transmission after different contact times.

**Contact**	**MDV strain**	**SPF chicken no.**									
		**1#**	**2#**	**3#**	**4#**	**5#**	**6#**	**7#**	**8#**	**9#**	**10#**
28 days	GX0101	93	217	221	427	–	269	94	310	192	281
	GX0101Δ*LTR*	–	–	114	169145	–	193	–	3982	–	105
35 days	GX0101	334	889	535	387	132	524	765	341	206	1074
	GX0101Δ*LTR*	–	–	397	231275	523	361	–	1139	–	197

*“–” represents the detection results are negative; the digits represent the copy number of virus. 1#–10# indicate the serial number of contact chickens.*

### Differentially Expressed MDV Genes Between GX0101 and GX0101Δ*LTR*

**FIGURE 4:**
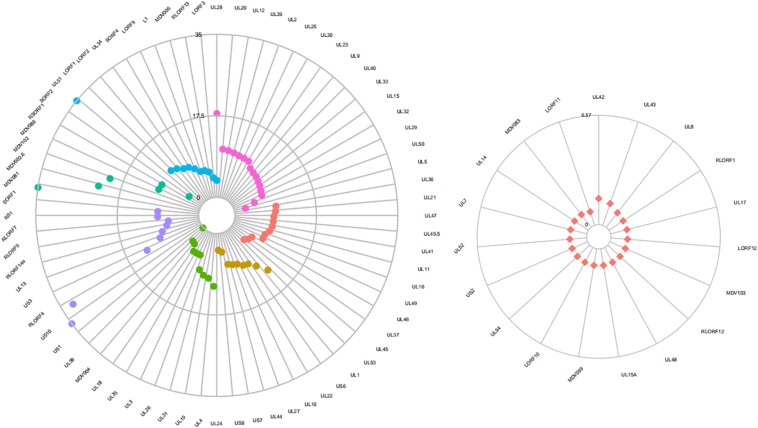
Differential expression of MDV genes between GX0101 and GX0101Δ*LTR.* Primary CEF cells were infected with GX0101 or GX0101Δ*LTR* at a dose of 1.5 × 10^5^ PFU per flask. The transcription profile of MDV viral genes in cell cultures was analyzed at 56 h post-infection using a 4 × 44 K Agilent custom oligo microarray (array ID: 042688). Four biological replicates were used in each group with dye balance. The transcription level of viral genes was compared between the two infectious groups. (a) Differentially expressed genes. Fuchsia represents virus replication gene and gene products; red represents genes coding tegument protein; yellow represents genes coding glycoprotein; green represents genes coding nucleocapsid protein; purple represents genes related to immune evasion, tumor development and/or pathogenesis; blue represents other genes encoding hypothetical proteins. (b) Genes with no significant difference in expression.

Five genes selected for validation by real-time RT-qPCR showed similar expression patterns as detected in microarray analysis (Table 7). Seventy-two genes were differentially expressed significantly between GX0101 and GX0101Δ*LTR* (Figure 4 and Supplementary Tables 1, 2). Compared to GX0101Δ*LTR*, the expression levels of 71 MDV genes in GX0101 were significantly up-regulated (GenBank No. JX844666). SORF2 gene had the greatest up-regulation fold change of 399, while the transcription level of UL38 in GX0101 was down-regulated with a fold change of 8.3. Among the proteins encoded by those differentially expressed genes, 16 were viral replication proteins, including UL28, UL20, UL12, UL39, UL2, UL25, UL30, UL23, UL9, UL40, UL33, UL15, UL32, UL29, UL50, and UL5. Eleven tegument proteins were UL36, UL21, UL47, UL49.5, UL41, UL11, UL16, UL49, UL46, UL37, and UL45. Nine of glycoproteins were UL53, UL1, US6, UL22, UL10, UL27, UL44, US7, and US8. Ten nucleocapsid proteins were UL24, UL4, UL19, UL31, UL26, UL3, UL35, UL18, MDV004, and UL38. Nine MDV genes and gene products were involved in immune evasion, tumor development and/or pathogenesis, including US1, US10, RLORF4, US3, UL13, RLORF14a, RLORF9, RLORF7, and RS1. Six hypothetical proteins were SORF1, MDV081, MDV002.6, MDV102.5, MDV086, and RSORF1. Eleven proteins, including SORF2, UL51, LORF1, LORF2, UL34, SORF4, LORF9, L1, MDV006, RLORF13, and LORF3, were other proteins with undefined function. The remaining 19 MDV genes were not differentially expressed between GX0101 and GX0101Δ*LTR*, including UL42, UL43, UL8, RLORF1, UL17, LORF12, MDV103, RLORF12, UL48, UL15A, MDV099, LORF10, UL54, US2, UL52, UL7, UL14, MDV083, and LORF11.

**TABLE 7:** Validation of microarray data by real-time RT-qPCR.

**Gene symbol**	**Primer sequences (5′–3′)**	**Fold change**	
		**Microarray**	**Real-time**
		**analysis**	**RT-PCR**
*SORF2*	F: TTTTGATTCCGTCTACCA	399.63	639.14
	R: AATACTCTAACAGCTCCTCC		
*US1*	F: GAGCCAGACCCGATACAC	85.42	115.36
	R: CACATAACCGAGCGACAT		
*US10*	F: CAACGGGCTGTGGAATAA	32.42	103.25
	R: CGTCTCCTGTTGGCGATT		
*UL24*	F: GTGGGAAGTAGGCTGTGA	11.37	13.64
	R:CAATCTGATCCTTGAGGC		
*UL50*	F: GTGGAGGTGGGATATGGG	4.64	3.63
	R: CGTTTCGTCTTCGGCAGT		

## Discussion

Marek’s disease has been prevalent among Chinese chicken flocks over the last 20 years (Cui et al., 2016; He et al., 2018). The incidence of MDV was highly heterogeneous in different regions and flocks because of the various geographical environments, different feeding patterns, and varieties of chicken breeds in China (Zhang et al., 2017). REV *LTR* could be integrated into the MDV genome at different sites following continuous passages of MDV on REV-infected CEF cells (Isfort et al., 1992). The recombination phenomenon between MDV and REV also exists in chickens (Woźniakowski et al., 2011).

To clarify the selective advantage of MDV GX0101 to becoming the more prevalent field strain in chicken flocks, the study established DF-qPCR, which specifically detects GX0101 and GX0101Δ*LTR* simultaneously to track and detect the viruses in the natural transmission model of MDV. The established qPCR system showed good specificity, in both the SF-qPCR system for detecting GX0101 or GX0101Δ*LTR*, respectively, and the DF-qPCR system for detecting the two viruses together though optimizing the reaction conditions (Figures 2, 3). Finally, DF-qPCR was established to detect viral copies of GX0101 and GX0101Δ*LTR* in a single tube accurately, which guaranteed the quantification of copies of the two viruses in a sample scientifically and reduced errors during operations.

Specific-pathogen-free chicks were inoculated with the same dose of GX0101 or GX0101Δ*LTR* to simulate MDV natural transmission under the same dose of spreading source. The DF-qPCR results showed that chicks could be infected with MDV at 14 dpi under direct contact with MDV-inoculated chicks, while GX0101Δ*LTR* was detectable until 28 dpi (Table 2). When the contact chicks were used as a spreading source, GX0101 was prevalent among all of the contact chicks after two generations of contact infection for 28 days, while GX0101Δ*LTR* was not detectable in any of the chicks (Supplementary Figure 4 and Tables 3, 4). GX0101 still could become the prevalent strain even though the initial infectious dose was significantly lower than GX0101Δ*LTR* (1/20) after the second round of passages (Supplementary Figure 5). Although GX0101Δ*LTR* could be detectable in a portion of contact chicks (60%) and even be the predominant strain in individual cases, GX0101 could be detectable in all of the contact chicks, with 40% of chicks infected with GX0101 alone (Tables 5, 6). Therefore, GX0101 possesses a higher transmission capacity and prevalent advantage than GX0101Δ*LTR*. It was demonstrated that the replication of GX0101Δ*LTR* virus was significantly lower than the GX0101 virus in the infected chickens in our previous study (Sun et al., 2010). The viremia level may be one of the important factors influencing horizontal transmission capacity, but other factors such as maturity of infectious viral particles, may also be involved.

The REV *LTR* possesses promoter and enhancer activity (Jones et al., 1996). Most of the viral genes were significantly up-regulated in GX0101 with an REV *LTR* insert as compared to GX0101Δ*LTR*, among which *SORF2* was maximally up-regulated. This is consistent with another recombinant MDV strain RM1 with an REV *LTR* insert produced on cell cultures, in which *SORF2* was also up-regulated (Isfort et al., 1992). However, the RM1 strain showed no tumorigenicity (Witter et al., 1997). A previous study also indicated that *SORF2* is non-essential for viral replication and tumor formation. The role of up-regulated *SORF2* in recombinant MDVs remains to be defined. Sixteen genes associated with viral replication were significantly up-regulated in GX0101 compared to GX0101Δ*LTR* (Figure 4). These viral genes mainly encoded DNA packaging protein, DNase-like protein, ribonucleotide reductase protein, uracil-DNA glycosylase, DNA polymerase processivity subunit-like protein, thymidine kinase, ori-binding protein, single-stranded DNA binding protein, dUTPase-like protein, and DNA helicase–primase associated protein. Among the 16 genes, UL28, UL20, UL12, and UL39 were up-regulated for more than 10 times. The function of MDV UL28 gene was not reported. It is speculated that product of UL28 is involved in the cleavage/packaging of herpesvirus DNA (Muylkens et al., 2010). UL28 of HSV-1 has been reported to be required for packaging of viral DNA, for formation of full capsids, and for expression of viral glycoproteins on the surface of virus-infected cells (Wills et al., 2006; Yang et al., 2017). The UL20 gene is conserved in all alphaherpesviruses, and the encoded protein is important for cytoplasmic virion morphogenesis and virus-induced cell fusion (Charles et al., 2014; Haque et al., 2016). Moreover, the HSV-1 UL20 is essential for viral replication (Carmichael and Wills, 2019). The MDV UL12 plays a major role in viral replication, but its precise role remains unknown (Previdelli et al., 2019). MDV UL39 encoded the large subunits of the ribonucleotide reductase (RR) enzyme, which is essential for replication in chickens, and important but not essential for viral replication in fibroblasts (Lee et al., 2013). Transcription of UL53, US1, US10, RLORF4, and UL24 was also up-regulated for more than 10 times. UL53-encoded glycoprotein K (gK) is essential for viral replication and is also involved in neurovirulence and immunomodulation in HSV-1 (Ren et al., 1994). The gK interacts with UL20 to form protein complex, and it has been suggested that this complex formation regulates virus entry and virus-induced cell fusion (Foster et al., 2008). UL1 encoded glycoprotein L (gL), which makes a hetero-oligomeric complex with gH (Yoshida et al., 1994). The gL/gH protein complex can modulate virus entry and cell-to-cell infection. US1 and US10 are important but not essential for viral replication, and closely related to formation of virus plaques (Parcells et al., 1994). RLORF4 is related to virulence, but its biological function remains unknown (Jarosinski et al., 2005). The function of UL24 is not reported in MDV. Our preliminary study confirms that the UL24-deleted MDV strain forms virus plaques slower than wild virus (unpublished data). In addition, UL13 and UL44, which were required for horizontal spread of MDV, were also up-regulated for more than six times (Jarosinski et al., 2007).

## Conclusion

We established DF-qPCR assay to quantify viral copies of GX0101 and GX0101Δ*LTR* with high specificity. Using the DF-qPCR, we simulated MDV natural transmission via continuous tracking of GX0101 and GX0101Δ*LTR* for the first time and illuminated that increased horizontal transmission of recombinant MDV due to REV *LTR* was the major competitive advantage of GX0101 being a prevalent strain. The differential transcription of viral genes between GX0101 and GX0101Δ*LTR* preliminarily revealed the molecular mechanism of increased horizontal transmission of MDV by REV *LTR*. The results are of great biological significance to study the recombination and evolution between different animal or even human viruses from various families and genera.

## Data Availability Statement

All datasets generated for this study are included in the article/Supplementary Material.

## Ethics Statement

The study protocol and all animal studies were approved by the Shandong Agricultural University Animal Care and Use Committee (SACUC Permission number: AVM01193-2) and performed in accordance with the “Guidelines for Experimental Animals” of the Ministry of Science and Technology (Beijing, China). Any bird deemed to have reached the humane endpoint was culled.

## Author Contributions

SS was involved in collection and assembly of the data, manuscript writing, and data analysis. ZQC, JC, and ZZC were involved in discussion and manuscript revision. YL, MY, and TZ performed the animal experiments. NC and RC conceptualized and designed the study, and were involved in the data analysis, manuscript revision, and final approval of the manuscript.

## Conflict of Interest

YL is employed by Zhaoqing Institute of Biotechnology Co., Ltd. The remaining authors declare that the research was conducted in the absence of any commercial or financial relationships that could be construed as a potential conflict of interest.

## Footnotes

1 http://www.r-project.org
