# Supplementary material for: Increased Horizontal Transmission of Recombinant Marek’s Disease Virus Due to Reticuloendotheliosis Virus Long Terminal Repeat Is the Major Competitive Advantage of the Virus Being a Prevalent Strain
Source: Front Microbiol. 2019 Dec 13;10:2842. doi: 10.3389/fmicb.2019.02842 (PMC6923665; doi:10.3389/fmicb.2019.02842)
Supplement: Supplementary file 1 [file Data_Sheet_1.pdf]

Supplementary Material

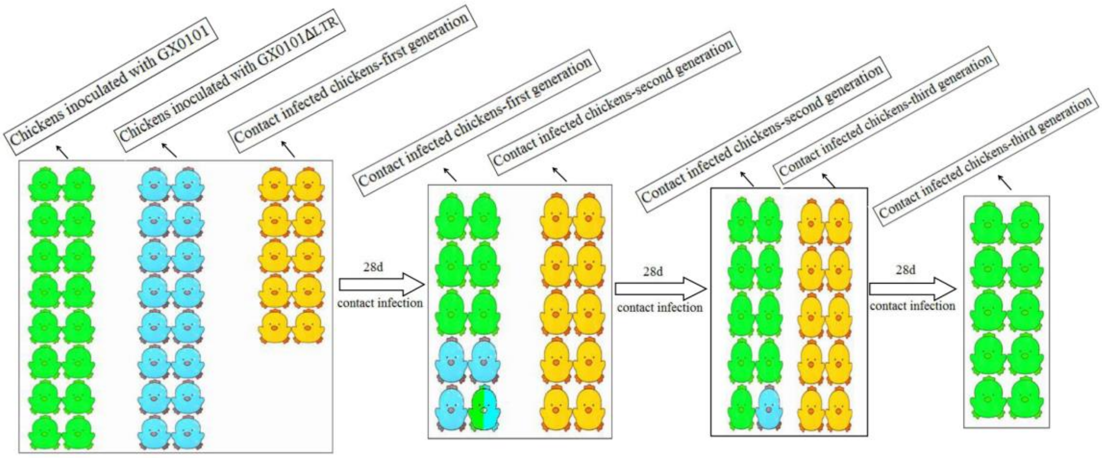

Figure 1 Sketch map of simulating MDV natural transmission in birds when the same dose of GX0101 or GX0101Δ*LTR* was inoculated.

Horizontal transmission capacity of GX0101 and GX0101Δ*LTR* were compared via successive contact infections by simulating MDV natural transmission in SPF chicks. Green birds were infected with GX0101; blue birds were infected with GX0101Δ*LTR*; yellow ones were uninfected birds.

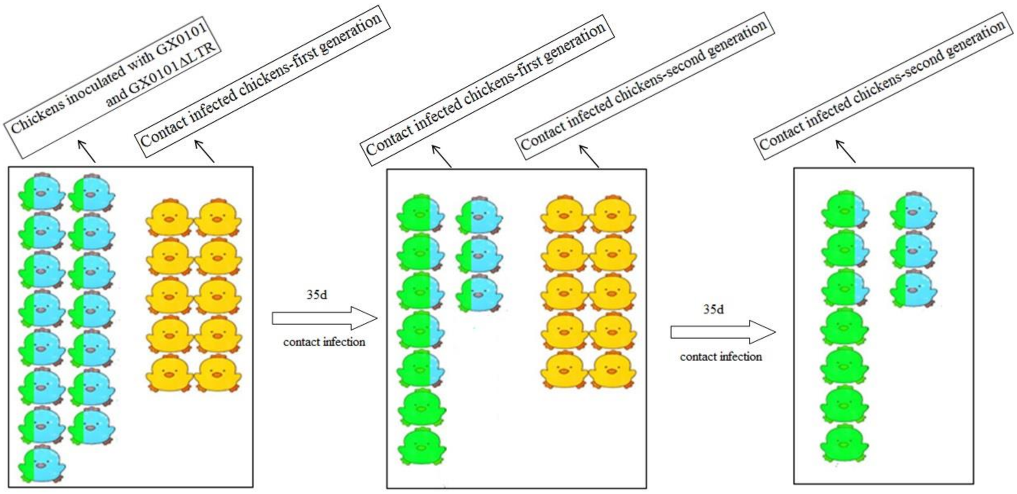

Figure 2 Sketch map of simulating MDV natural transmission in birds when different dose of GX0101 or GX0101Δ*LTR* was inoculated.

Fifteen SPF birds were inoculated with 100 PFU of GX0101 or 2000 PFU of GX0101ΔLTR by the IA route. Horizontal transmission capacity of GX0101 and GX0101Δ*LTR* in birds was compared via successive contact infections. Green birds were infected with GX0101; blue birds were infected with GX0101Δ*LTR*; yellow ones were uninfected birds.

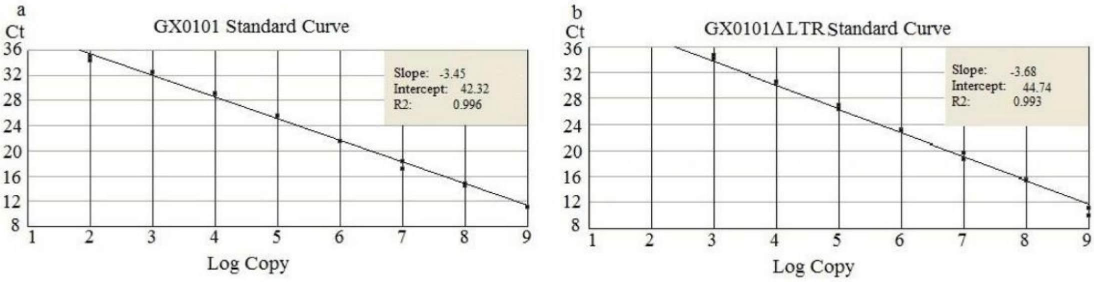

Figure 3 Standard curves of GX0101 (a) and GX0101Δ*LTR* (b)

The Ct values of GX0101 (a) and GX0101Δ*LTR* (b) from 10^9 to 10^1 copies were detected. Log Copy: Log (Copy number of the virus).

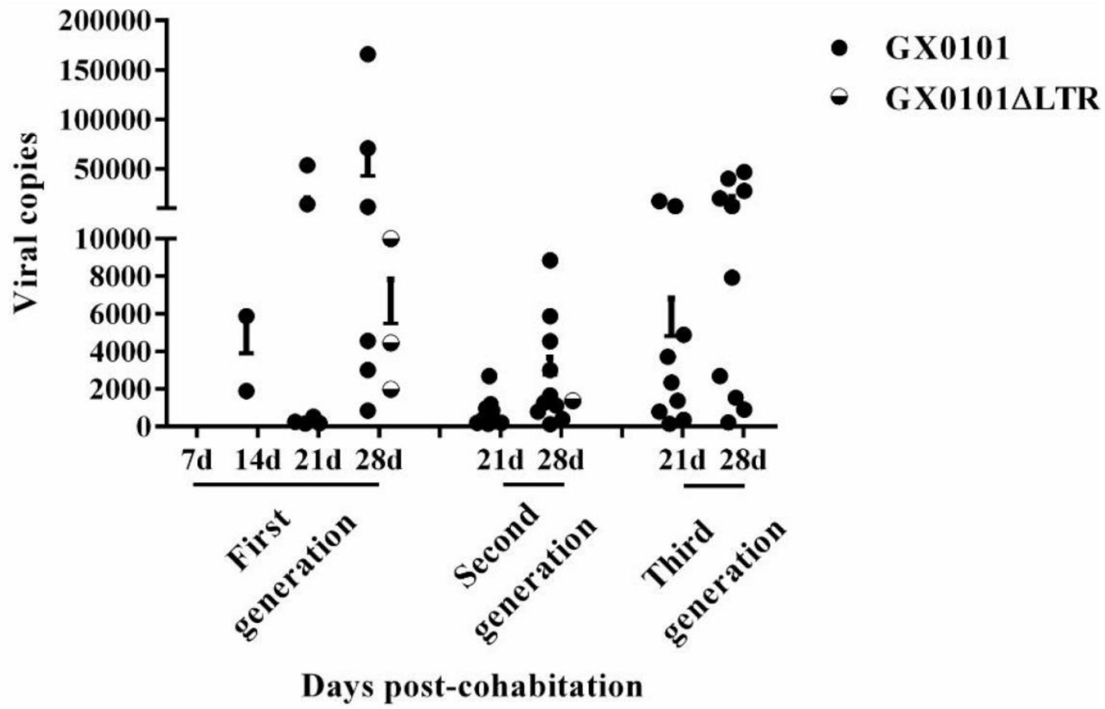

Figure 4 GX0101 has significant advantage of horizontal transmission over GX0101ΔLTR when inoculating with the same dose

SPF chicks were inoculated with 1000 PFU of GX0101 or GX0101Δ*LTR*. Horizontal transmission capacity of GX0101 and GX0101Δ*LTR* were compared via successive contact infections by simulating MDV natural transmission. Viral copies of GX0101 and GX0101Δ*LTR* in the first round of contact chicks were determined by detecting the DNA of the feather tips using DF-qPCR at 7, 14, 21 and 28 days post-inoculation, respectively. Viral copies in the second and the third rounds of contact chicks were determined after 21 and 28 days cohabitation, respectively.

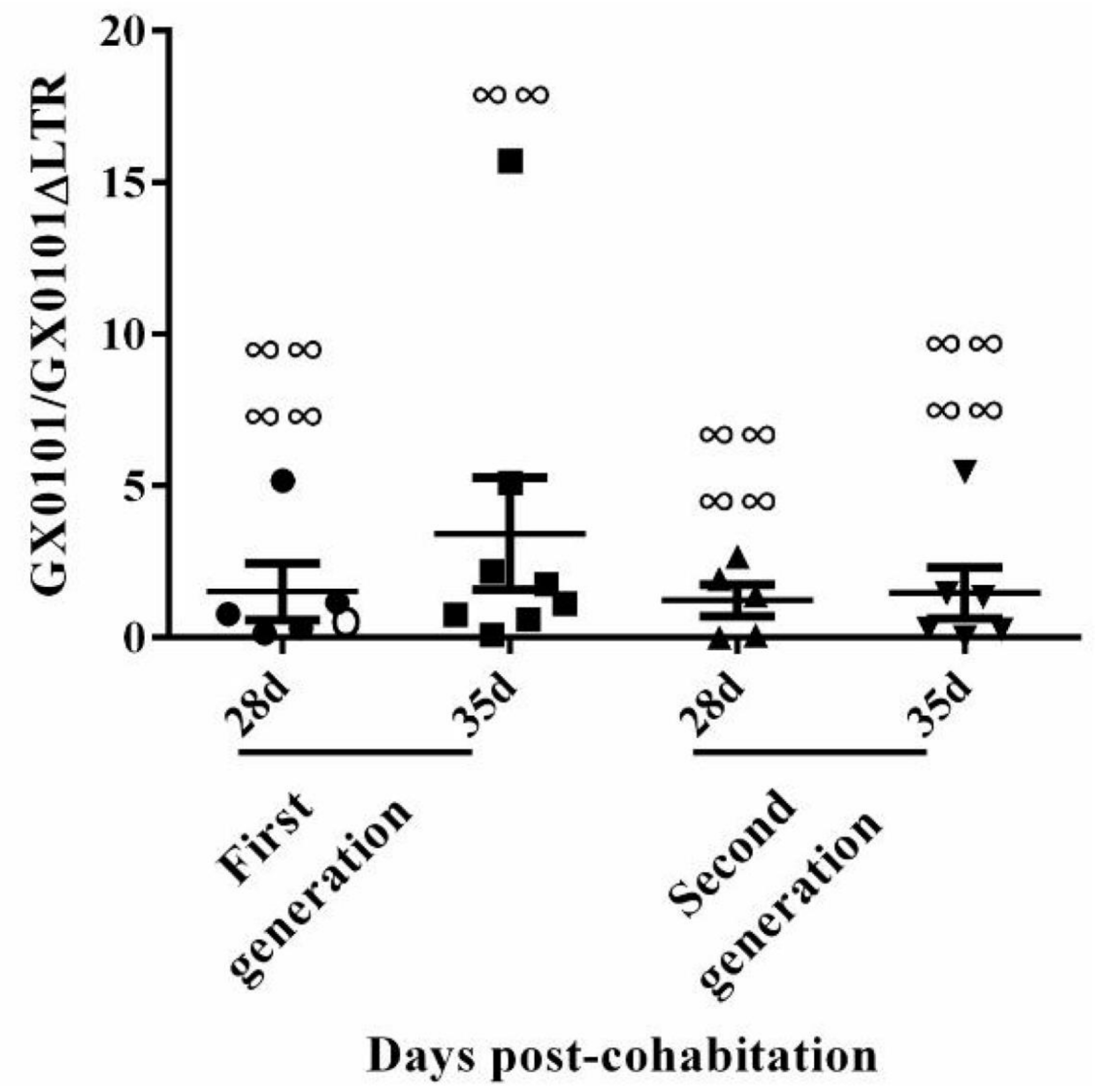

Figure 5 GX0101 still has significant advantage of horizontal transmission over GX0101ΔLTR when a lower dose was inoculated

SPF chicks were inoculated with 100 PFU of GX0101 or 2000 PFU of GX0101Δ*LTR*. Viral copies of the two viruses in the first and second rounds of contact chicks were determined after 28 and 35 days cohabitation, respectively.The ratio of viral copies (GX0101/GX0101Δ*LTR*) in feather follicles was calculated. ∞: GX0101 was only detectable; ○: GX0101Δ*LTR* was only detectable; ●, ■▲▼: The ratio of viral copies (GX0101/GX0101Δ*LTR*) in feather follicles was calculated at different time points.
