# Supplementary material for: Increased Horizontal Transmission of Recombinant Marek’s Disease Virus Due to Reticuloendotheliosis Virus Long Terminal Repeat Is the Major Competitive Advantage of the Virus Being a Prevalent Strain
Source: Front Microbiol. 2019 Dec 13;10:2842. doi: 10.3389/fmicb.2019.02842 (PMC6923665; doi:10.3389/fmicb.2019.02842)
Supplement: Supplementary file 2 [file Data_Sheet_2.doc]

Table 1 Differentially expressed MDV genes between GX0101 and GX0101∆*LTR*.

| Probe ID | Gene Name | Fold change | Adjusted P Value |
| --- | --- | --- | --- |
| Virus replication | | | |
| 021216 | UL28, DNA packaging protein-like protein (MDV041) | 18.00 | 0.00095 |
| 021208 | UL20, transmembrane protein-like protein, virus egress (MDV032) | 10.4 | 3.05E-05 |
| 021199 | UL12, DNase-like protein (MDV024) | 10.25 | 4.34E-05 |
| 021227 | UL39, ribonucleotide reductase large subunit-like protein (MDV052) | 10.03 | 0.00084 |
| 021189 | UL2, uracil DNA glycosylase-like protein (MDV014) | 9.77 | 0.00042 |
| 021213 | UL25, DNA packaging-like protein (MDV037) | 9.70 | 0.00011 |
| 021218 | UL30, DNA polymerase catalytic subunit-like protein (MDV043) | 9.58 | 8.97E-05 |
| 021211 | UL23, thymidine kinase-like protein (MDV036) | 8.49 | 0.00028 |
| 021196 | UL9, ori binding protein-like protein (MDV021) | 8.16 | 0.00063 |
| 021228 | UL40, ribonucleotide reductase large subunit-like protein (MDV053) | 7.95 | 0.0016 |
| 021221 | UL33, DNA packaging (MDV045) | 7.58 | 8.59E-05 |
| 021205 | UL15, DNA packaging protein-like protein (MDV027) | 7.27 | 0.00028 |
| 021220 | UL32, DNA packaging protein-like protein (MDV046) | 6.94 | 6.54E-06 |
| 021217 | UL29, single stranded DNA binding protein-like protein (MDV042) | 6.69 | 0.00082 |
| 021239 | UL50, dUTPase-like protein (MDV063) | 4.64 | 0.018 |
| 021192 | UL5, DNA helicase-primase associated protein-like protein (MDV017) | 2.44 | 6.75E-05 |
| Tegument | | | |
| 021224 | UL36, large tegument protein-like protein (MDV049) | 8.97 | 0.00010 |
| 021209 | UL21, tegument protein-like protein (MDV033) | 8.73 | 0.00054 |
| 021235 | UL47, tegument phosphoprotein-like protein (MDV060) | 8.36 | 4.94E-05 |
| 021238 | UL49.5, envelope/tegument protein-like protein (MDV064) | 8.30 | 0.00011 |
| 021229 | UL41, virion host shutoff protein-like protein (MDV054) | 8.25 | 9.34E-06 |
| 021198 | UL11, myristylated tegument protein-like protein (MDV023) | 7.93 | 0.0040 |
| 021203 | UL16, tegument protein-like protein (MDV028) | 7.21 | 0.0019 |
| 021237 | UL49, tegument phosphoprotein-like protein (MDV062) | 7.19 | 0.0011 |
| 021234 | UL46, tegument phosphoprotein-like protein (MDV059) | 4.97 | 0.00031 |
| 021225 | UL37, tegument protein-like protein (MDV050) | 4.58 | 0.00044 |
| 021233 | UL45, envelope/transmembrane protein-like protein, cell fusion (MDV058) | 3.93 | 6.80E-05 |
| Glycoprotein | | | |
| 021242 | UL53, glycoprotein K-like protein (MDV067) | 12.18 | 6.14E-05 |
| 021188 | UL1, virion surface glycoprotein L-like protein (MDV013) | 9.90 | 3.39E-05 |
| 021270 | US6, membrane glycoprotein D-like protein (MDV094) | 8.52 | 0.0012 |
| 021210 | UL22, envelope glycoprotein H-like protein (MDV034) | 8.25 | 2.11E-05 |
| 021197 | UL10, viron membrane glycoprotein M-like protein (MDV022) | 7.47 | 0.0013 |
| 021215 | UL27, virion membrane glycoprotein B-like protein (MDV040) | 7.20 | 0.00016 |
| 021232 | UL44, virion membrane glycoprotein C-like protein (MDV057) | 6.88 | 0.00028 |
| 021271 | US7, membrane glycoprotein I-like protein (MDV095) | 4.05 | 0.0013 |
| 021272 | US8, membrane glycoprotein E-like protein (MDV096) | 3.61 | 0.034 |
| Nucleocapsid | | | |
| 021212 | UL24, nuclear protein (MDV035) | 11.37 | 0.0039 |
| 021191 | UL4, nuclear protein-like protein (MDV016) | 9.72 | 4.96E-05 |
| 021207 | UL19, major capsid protein-like protein (MDV031) | 9.28 | 0.00059 |
| 021219 | UL31, nuclear phosphoprotein-like protein (MDV044) | 8.38 | 0.0028 |
| 021214 | UL26, capsid maturational protease, scaffold protein (MDV038) | 5.33 | 0.013 |
| 021190 | UL3, nuclear phosphoprotein-like protein (MDV015) | 5.31 | 0.017 |
| 021223 | UL35, capsid protein-like protein (MDV048) | 5.30 | 0.0015 |
| 021206 | UL18, nucleocapsid protein-like protein (MDV030) | 3.91 | 0.0032 |
| 021174 | 23kDa nuclear protein (MDV004) | 3.66 | 0.00020 |
| 021226 | UL38, capsid protein-like protein (MDV051) | 0.12 | 0.00038 |
| MDV genes and gene products involved in immune evasion, tumor development and/or pathogenesis | | | |
| P039340 | US1, phosphoprotein ICP22-like protein (MDV088) | 85.42 | 1.41E-06 |
| P039336 | US10, viron membrane glycoprotein M-like protein (MDV022) | 32.42 | 0.0029 |
| 021171 | RLORF4 (MDV003.4) | 12.87 | 0.0027 |
| 021268 | US3, serine threonine protein kinase-like protein (MDV092) | 9.22 | 4.76E-05 |
| 021200 | UL13, serine/threonine protein kinase (MDV025) | 8.32 | 3.08E-06 |
| 021249 | RLORF14a, 38 kDa phosphoprotein, pp38 (MDV073) | 7.08 | 1.20E-05 |
| 021176 | RLORF9, 14 kDa lytic protein C (MDV005.7) | 6.59 | 0.026 |
| 021173 | RLORF7, meq protein (MDV005) | 8.84 | 5.85E-05 |
| 021259 | RS1, immediate-early gene transactivator ICP4-like protein (MDV084) | 8.83 | 0.0011 |
| Hypothetical protein | | | |
| P039338 | SORF1, hypothetical protein | 106.01 | 7.29E-06 |
| 021257 | hypothetical protein (MDV081) | 22.30 | 0.00011 |
| 100153 | hypothetical protein (MDV002.6) | 20.38 | 1.65E-05 |
| 021276 | hypothetical protein (MDV102.5) | 9.78 | 0.00045 |
| 021273 | hypothetical protein (MDV086) | 9.62 | 4.48E-07 |
| 021258 | RSORF1 (MDV102) | 3.31 | 0.0084 |
| Other protein | | | |
| P039334 | SORF2, binds growth hormone (MDV087) | 399.63 | 2.18E-06 |
| 021240 | UL51, virion phophoprotein-like protein (MDV065) | 9.88 | 0.0014 |
| 021183 | LORF1 (MDV009) | 9.3 | 4.04E-05 |
| 021185 | LORF2 (MDV008.4) | 8.43 | 6.76E-05 |
| 021222 | UL34, membrane phosphoprotein-like protein (MDV047) | 8.08 | 0.00018 |
| 021269 | SORF4 (MDV093) | 7.18 | 5.64E-05 |
| 021244 | LORF9 (MDV069) | 6.14 | 0.00092 |
| 021285 | L1, transcript identified in MD lymphoblastoid chicken cells (MDV078.1) | 6.09 | 1.57E-06 |
| 021178 | 14 kDa lytic phase protein splice variant A (MDV006) | 5.55 | 0.0011 |
| 021184 | RLORF13, binds C1q-binding protein (MDV009.5) | 4.23 | 0.00021 |
| 021187 | LORF3 (MDV012) | 3.62 | 0.0081 |

Table 2 Genes with no significant difference in expression between GX0101 and GX0101∆*LTR*.

| Probe ID | Gene ID | Fold change | Adjusted P Value |
| --- | --- | --- | --- |
| 021230 | UL42, DNA polymerase (MDV055) | 1.57 | 0.41934 |
| 021231 | UL43,probable membrane protein (MDV056) | 1.38 | 0.188475 |
| 021195 | UL8, DNA helicase-primase associated protein (MDV020) | 1.10 | 0.561061 |
| 021169 | RLORF1, Arg-rich protein (MDV079) | 1.02 | 0.956936 |
| 021204 | UL17, tegument protein; DNA packaging (MDV029) | 1.01 | 0.346022 |
| 021248 | LORF12 (MDV072.8) | 1.01 | 0.3468 |
| 021277 | MDV103 | 1.01 | 0.35781 |
| 021252 | RLORF12, binds growth-related translationally-controlled tumor protein (MDV074) | 1.01 | 0.359002 |
| 021236 | UL48, tegument immediate early protein VP16 (MDV061) | 1.01 | 0.359861 |
| 021202 | UL15A | 1.01 | 0.361628 |
| 021274 | MDV099 | 1.01 | 0.369241 |
| 021246 | LORF10, similar to VZV ORF2 (MDV071) | 1.01 | 0.368117 |
| 021243 | UL54, ICP27-like protein; posttranslational gene~regulation (MDV068) | 1.01 | 0.368117 |
| 021267 | US2 (MDV091) | 1.01 | 0.375479 |
| 021241 | UL52, DNA helicase-primase associated protein (MDV066) | 1.01 | 0.39165 |
| 021194 | UL7, capsid protein; DNA packaging/cleavage (MDV019) | 1.01 | 0.396059 |
| 021201 | UL14, minor tegument protein (MDV026) | 1.00 | 0.945608 |
| 021275 | MDV083 | 0.96 | 0.735292 |
| 021247 | LORF11 (MDV072) | 0.86 | 0.631011 |
